# Interaction of Hepatitis C virus proteins with pattern recognition receptors

**DOI:** 10.1186/1743-422X-9-126

**Published:** 2012-06-22

**Authors:** Muhammad Imran, Yasir Waheed, Sobia Manzoor, Muhammad Bilal, Waseem Ashraf, Muhammad Ali, Muhammad Ashraf

**Affiliations:** 1 Atta Ur Rahman school of Applied Biosciences, National University of Sciences and Technology, Islamabad 44000, Pakistan

**Keywords:** Hepatitis C virus, Toll-like receptors, Anti-viral pathways

## Abstract

Hepatitis C virus (HCV) is an important human pathogen that causes acute and chronic hepatitis, cirrhosis and hepatocellular carcinoma worldwide. This positive stranded RNA virus is extremely efficient in establishing persistent infection by escaping immune detection or hindering the host immune responses. Recent studies have discovered two important signaling pathways that activate the host innate immunity against viral infection. One of these pathways utilizes members of Toll-like receptor (TLR) family and the other uses the RNA helicase retinoic acid inducible gene I (RIG-I) as the receptors for intracellular viral double stranded RNA (dsRNA), and activation of transcription factors. In this review article, we summarize the interaction of HCV proteins with various host receptors/sensors through one of these two pathways or both, and how they exploit these interactions to escape from host defense mechanisms. For this purpose, we searched data from Pubmed and Google Scholar. We found that three HCV proteins; Core (C), non structural 3/4 A (NS3/4A) and non structural 5A (NS5A) have direct interactions with these two pathways. Core protein only in the monomeric form stimulates TLR2 pathway assisting the virus to evade from the innate immune system. NS3/4A disrupts TLR3 and RIG-1 signaling pathways by cleaving Toll/IL-1 receptor domain-containing adapter inducing IFN-beta (TRIF) and Cardif, the two important adapter proteins of these signaling cascades respectively, thus halting the defense against HCV. NS5A downmodulates the expressions of NKG2D on natural killer cells (NK cells) via TLR4 pathway and impairs the functional ability of these cells. TLRs and RIG-1 pathways have a central role in innate immunity and despite their opposing natures to HCV proteins, when exploited together, HCV as an ever developing virus against host immunity is able to accumulate these mechanisms for near unbeatable survival.

## Introduction

HCV infection is a major cause of acute hepatitis and chronic liver disease. HCV was first identified in 1989 [1]. It is classified as a member of the *Hepacivirus* genus within the family *Flaviviridae.* More than 200 million people are living with HCV, which covers about 3.3% of the world’s population [2]. It is also estimated that three to four million people are infected with HCV each year [3]. There are 6 genotypes of HCV, 52 subtypes within these genotypes, as well as diverse population of quasispecies within each infected individual. The source of this variation, like that of other RNA viruses, is the high mutation rate of its error prone RNA polymerase [4]. The HCV genome is approximately 9.6 kb and encodes an approximately 3000 amino acids long polyprotein. Complicated quasispecies and frequent mutation of viral genome have also emerged. The large HCV polyprotein is cleaved by the host and viral proteases to generate at least 10 proteins, including four structural proteins (core protein, two envelope proteins, E1, E2, and p7 ion channel). The six non-structural proteins include NS2-NS3-NS4A-NS4B-NS5A-NS5B-COOH [5]. These proteins not only have a role in viral replication but also have an important role in cellular function. Although there have been several explanations suggested for HCV infection, replication in targeted cells and escape from the immune system, the actual mechanism is still not understood [6]. Patients in the advanced stages of chronic liver disease in consequence of viral infection or alcohol abuse show higher susceptibility to microbial infections and are considered to be immune compromised hosts [7]. Impaired activation of TLR signaling may also have a role in the susceptibility to infections in patients with liver cirrhosis [8]. Innate immunity is made possible by a network of germ-line encoded pattern-recognition receptors (PRRs), which senses pathogen-associated molecular patterns (PAMPs) on invading microbes and activate immunological responses. PRRs include the Nod-like receptors (NLRs), RIG-like receptors (RLRs), Toll-like receptors (TLRs) and the recently identify cytosolic DNA receptors [9-12]. TLRs are evolutionarily conserved structures. They were initially demonstrated in the fruit fly *Drosophila melanogaster* as an element governing its body’s longitudinal growth as well as an anti-fungal agent. Soon after that structures homologous to TLRs were also characterized in higher animals, including humans. They can recognize a variety of molecules that are present in various pathogens and are essential for their growth and survival. PAMPs are divided into subfamilies on the basis of their broad specificity, ranging from TLR1 to TLR11 in human. TLRs are mainly expressed on the cell surface (TLRs1, 2, 4, 5, 6, and 10). However, some TLRs such as TLR3, 7, 8, and 9 are located intracellularly [13]. Mechanisms responsible for liver damage associated with chronic HCV infection remain incompletely understood, although increasing evidence points to immunological rather than direct viral effects [14-17]. Several bacterial infections such as sepsis and cellulitis are more common in HCV-infected patients than in those without HCV infection [7,18,19]. As TLR mediated proinflammatory cytokine responses are necessary for host defense against bacteria [20], it is likely that chronic HCV infection generates an immune environment in which TLR-mediated proinflammatory cytokine production is impaired after exposure to bacterial antigens. In this review article we focused on the interaction of HCV proteins with TLRs & RIG1 and how these interactions are exploited by HCV to escape from the host innate immune system.

### HCV core protein and TLRs

The HCV core protein possesses many functions. It interacts with many cellular proteins and signal transduction pathways of the host cell. Its main function is to form the capsid shell that houses and protects the HCV genomic RNA while the virus passes from one cell to another or from one person to another [21]. When human embryonic kidney 293 cells (HEK293) and human monocytic cell line, Mono Mac 6 (MM6) cell lines were stimulated by HCV core protein; there was an induction of IL-6 and IL-8 via TLR2 pathway. MyD88-deficient splenocytes fail to produce IL-6 after stimulation with the core protein further confirming the role of core protein in TLR2 signaling pathway as MyD88 is a downstream effector molecule of the TLR2 signaling cascade [22]. Thus, core protein is a specific activator of the TLR2-MyD88 signaling cascade, it causes the activation of TLR2 on antigen-presenting cells (APCs) to induce cytokines that are produced in response to nuclear translocation of nuclear factor kappa B (NFκB) subunits [21,22]. Although the ligation of TLRs on APCs induce proinflammatory responses, pre-exposure to TLR2 or TLR4 ligands desensitize APCs to subsequent stimulation by TLRs [23-26]. Decreased production of TNFα in response to challenge with a TLR ligand with which the cells were pre-treated is defined as “homotolerance”, while decreased activation to a TLR ligand that does not share homology with the pretreatment ligand is called “heterotolerance” [27]. TLR tolerance has a significant role in protecting from hyperactivation of the immune system and can be overcome by conditioning of innate immune cells with cytokines, including interferons and growth factors [27-29]. Exposure of MM6 cells to core protein also induces homotolerance of TLR2 and cross tolerance with TLR4 after subsequent stimulation with TLR ligands. The induction of cross-tolerance by exposure to core protein is impaired by blockage of the TLR2 pathway, because IL-6 production is significantly reduced in MM6 cells treated with an anti-TLR2 monoclonal antibody [22].

The production of IL-6 and IL-8 by monocytes isolated from HCV-infected patients are also significantly decreased as compared to that of healthy control subjects when cells are stimulated with core protein, peptidoglycan Pam3CSK4, or lipopolysaccharide (LPS). The continuous activation of TLR2 by core protein causes decreased cytokine responses to TLR ligands in monocytes from HCV-infected patients [30]. There is no considerable correlation between serum levels of the core antigen and TLR-induced IL-6 or IL-8 production. This suggests that the serum concentration of the core antigen is not responsible for hyporesponsiveness to TLR ligands during chronic HCV infection. Other viral proteins such as NS3/4A and host factors may also be involved in the generation of hyporesponsiveness to TLR ligands. Furthermore, there is no association between patient age and the production of proinflammatory cytokines in patients with chronic HCV infection [8]. Reduced production of IL-6 by peripheral blood mononuclear cells (PBMCs) from chronic HCV patients is associated with decreased platelet counts and prolonged prothrombin time (PT). As these parameters are very sensitive markers of liver function [7], it signifies that liver dysfunction is associated with hyporesponsiveness to TLR ligands in patients with chronic HCV infection. Both viral and host factors have a role in the generation of impaired responses to TLR ligands during chronic HCV infection. Platelet counts and PT can be used as markers to determine TLR responses in patients with chronic HCV infection [31]. Pre-activation with core protein causes no significant alteration in the expression of costimulatory molecules and TLRs, CD80, CD86, TLR2, or TLR4 in MM6 cells. The expression of CD80 and CD86 is not changed by treatment with core protein after re-stimulation with core protein or lipopolysaccharide. It is improbable that the inhibition of TLR responses by core protein pre-stimulation is due to the induction of apoptotic cell death because the stimulation of MM6 cells with core protein has no effect on the percentage of Annexin V + apoptotic cells [22].

### Involvement of TLR2 corecptors

There is also a significant role for TLR2 coreceptors in cellular activation by the core protein in human and mouse cells. The absence of TLR1 or TLR6 had a striking negative effect on HCV core stimulation, suggesting the role of both of these TLR2 coreceptors. Nonetheless, selective silencing of only one coreceptor did not result in the complete loss of cytokine induction by the HCV ligands. The knockout mouse model demonstrates that HCV core and NS3 use the TLR2/TLR6 complex. The only minimal inhibition of HCV core induced TNF-α in TLR1-/- recommends that in mice, recognition or activation of HCV core may not involve TLR1. As HCV does not infect mice and their TLRs have slightly different sequences than humans, it is not astonishing that there may be some differences in the ligand activation between these two TLR2 coreceptors. There is a significant reduction in TNF-α in the HCV protein-stimulated TLR6 knockout macrophages but the residual cytokine production remained active. So, it means that there is the possibility of an alternate use of TLR1 or the probability of the use of another TLR2 coreceptor, such as CD36 (dectin-1) [32,33] or CD14 [34] in human beings. Use of TLR1 or TLR6 as TLR2 coreceptors in macrophage activation by HCV core protein supports a potential for broad range identification and cell activation by these proteins.

### Evasion from the innate immune system

**Figure 1:**
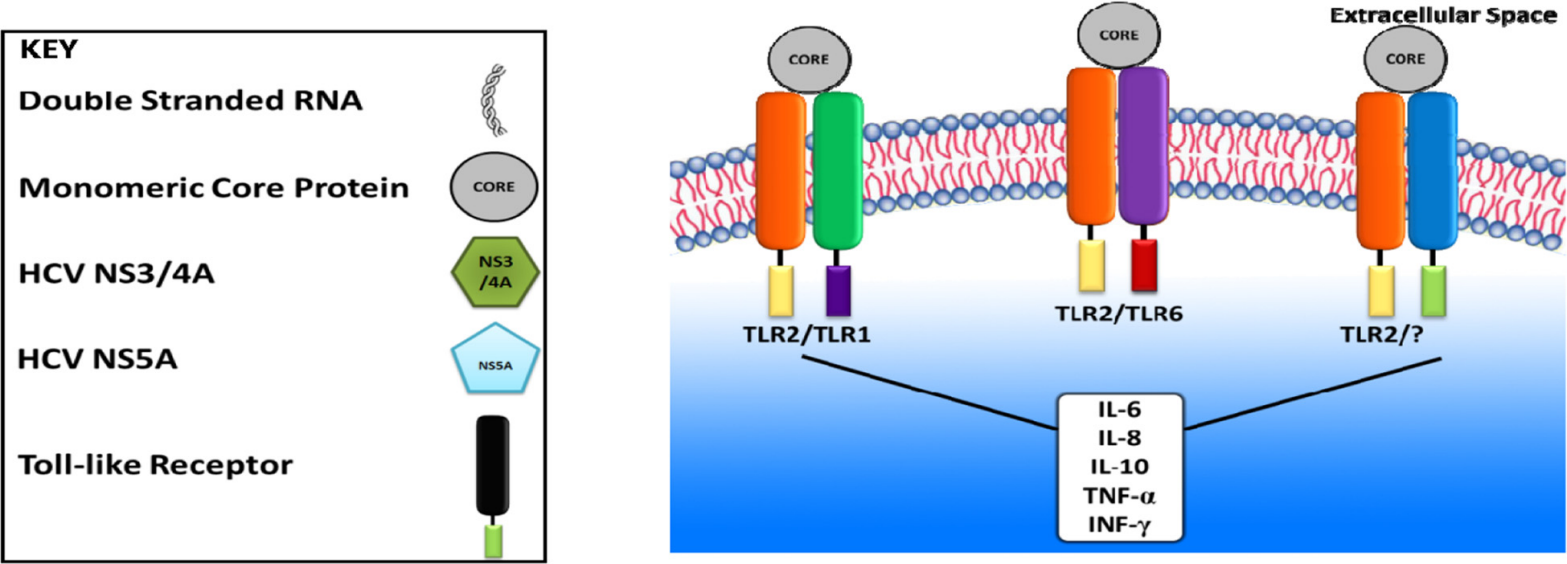
HCV monomeric core protein interacts with TLR2 and utilizes TLR1, TLR6 or some other receptor as a coerceptor.

HCV core protein in monomeric form is sensed by TLR2 as shown in Figure 1. On the contrary, neither recombinant nor serum derived infectious HCV results in efficient activation of TLR2 signaling. Target cell lines including human dendritic cells [35] and hepatocytes express TLR2 at noteworthy levels. It has been suggested that HCV core sensed by TLR2 appears to be absent in enveloped viral particles. Two mechanisms may explain this evasion: (a) In intact viral particles, the core protein may take conformation that might not be acknowledged by TLR2; (b) the envelope glycoproteins in infectious virions prejudice HCV core sensing by TLR2. It has been shown that denaturation of monomeric core protein by heat treatment completely abolishes TLR2 sensing, supporting the first hypothesis of conformation dependent sensing of core protein. The second hypothesis, impairment of core sensing by the HCV envelope is supported by the finding that the recombinant envelope glycoproteins and patient-derived HCV are not capable of stimulating TLR2 signaling pathway [36]. HCV entry into host cells is a complex process involving several binding and entry factors that ultimately lead to decapsidation of the virus inside the endosomal compartments and release of viral RNA into the cytoplasm. As the monomeric core protein, but not core as part of a nucleocapsid or subviral particle interacts with TLR2, it is possible that HCV particles are not sensed by cell surface expressed TLR2 during the steps of binding and entry. In vivo, TLR2 sensing and activation is most likely to be activated by core protein that is resulted from degraded nucleocapsids or unassembled core protein which are released from disintegrated infected hepatocytes at distinct sites of the infected host. As recent studies have demonstrated the evidence for intracellular expression of TLRs [37], it is more feasible that core-TLR2 interaction and activation may occur following viral uncoating or production of monomeric protien in an intracellular compartment. Thus, only monomeric core protein but not infectious viral particles is sensed by TLR2. Impairment of core-TLR interaction in infectious particles may have role to escape from innate antiviral immune responses and facilitate persistence of HCV infection [36].

### Evasion from the adaptive immune system

#### Defect in Th17 differentiation

Chronic exposure to the core protein leads to the development of APCs with a limited ability to drive Th17 differentiation. IL-17 (but not IFN-γ) production by allogeneic naïve CD4+ T cells is clearly reduced when T cells are cocultured with monocytes from HCV-infected patients and with TLR ligands. This discriminating impairment of the adaptive IL-17 response can be explained by profiles of cytokine production by these monocytes. IL-6 production induced by core protein and TLR ligands is significantly decreased in monocytes from HCV-infected patients as compared to healthy control subjects; whereas IL-12p40 production is comparable in monocytes from both populations. As IL-6 and IL-12 play an important role for Th17 and Th1 differentiation, respectively [32,38,39], the defective IL-17 response in allogeneic CD4+ T cells may be due to impaired IL-6 production by APCs from HCV-infected patients. Chronic exposure to the core protein seems to impair the adaptive IL-17 response (through the development of APCs with a limited ability to produce IL-6 after stimulation with TLR ligands) without having any effect on adaptive interferon gamma (IFN-γ) or transforming growth factor beta (TGF-β) responses [40].

#### Defect in PDC modulation

HCV Core protein via TLR2 plays an important role in the modulation of plasmacytoid cell (PDC) functions as an anti-TLR2 antibodies (Abs) partially prevent the inhibitory effect of HCV core protein on TLR9-triggered IFN-α production. Thus core protein exploits TLR2 pathway to reduce IFN-α production for its survival. IL-10 [41] and TNF-α [42] are induced by TLR2 pathway. Antibody neutralization of IL-10 and TNF-α repaired IFN-α production in TLR9 plus HCV core stimulated PBMCs. Additionally, rIL-10 and TNF-α repressed both TLR9-induced IFN-α production and PDC apoptosis in a dose dependent manner. It means that the microenvironment, including the presence of cytokines and chemokines, is fundamental for PDC function [43-46]. ILs 3, 4, 7, and 15 support IFN-α production of virus-infected cells whereas TNF-α and IL-10 inhibit it [43]. Under normal regulation TLR tolerance confines TNF-α production and it is protected against excessive immune activation and its harmful effects [47]. These protective mechanisms are deregulated in HCV infected patients. HCV patient’s monocytes fail to set homo and heterotolerance to pro-inflammatory cytokine involving TLR ligands, including TLR2/TLR1, TLR2/TLR6, TLR4, TLR3 and TLR7/8. HCV core protein [23,48] activates and induces tolerance in normal but not in chronic HCV monocytes and disturbs the regulation of cytokines.

### NS3/4A and escape from the innate immune system

The role played by TLR3 in HCV infection is much less convinced. NS3/4A is a non covalent enzyme complex that possesses RNA helicase as well as protease activity. It leads to post-translational cleavage of the polyprotein expressed by this positive-strand RNA virus [49]. One of the most important host responses to virus infection is the production of chemokines and antiviral cytokines such as IFN-α and IFN-β. Virus-induced IFN production is added by positive feedback mechanisms via type I IFNs [50]. The initial step for the stimulation of cytokine response in RNA virus infection is cellular activation of dsRNA receptor systems, Toll-like receptor 3 (TLR3) [51,52] and retinoic acid inducible gene-I (RIG-I) [53]. These two pathways lead to the activation of IκB kinase (IKK) α/β/γ complex and IKK-like kinases e.g. IKKϵ and TANK binding kinase 1 (TBK1) [53-57]; which mediate the activation and nuclear translocation of NFκB and interferon regulatory factor 3 (IRF3) [58,59]. Inside the nucleus; IRF3, NFκB and activator protein 1 (AP-1), transcription factors stimulate type I IFN and proinflammatory cytokine genes expression. Many viruses have evolved the strategy to impede the effector mechanisms induced through these pathways [60], but viral interference with the significant proximal receptor interactions has not yet been depicted. NS3/4A appears to mediate proteolysis of a cellular protein within an antiviral signaling pathway upstream of IRF-3. IFN-α (α1), IFN-β and IFN-λ (λ1) genes are extremely sensitive to the inhibitory effect of NS3/4A. There is also an inhibitory effect of NS3/4A on other cytokine/chemokine gene promoters such as IFN-β, CCL5/RANTES,CXCL10/ IP-10, CXCL8/IL-8, TNF-α and IFN-α4. Thus, NS3/4A protein is not only an effective antagonist of the IFN-β promoter but also of other cytokine/chemokine promoters. Inhibition of IRF-3 activation requires only NS3/4A protease activity and is abrogated by a specific, peptido-mimetic protease inhibitor, SCH6 [61].

#### Disruption of TLR3 and RIG-I pathways by HCV NS3/4A

**Figure 2:**
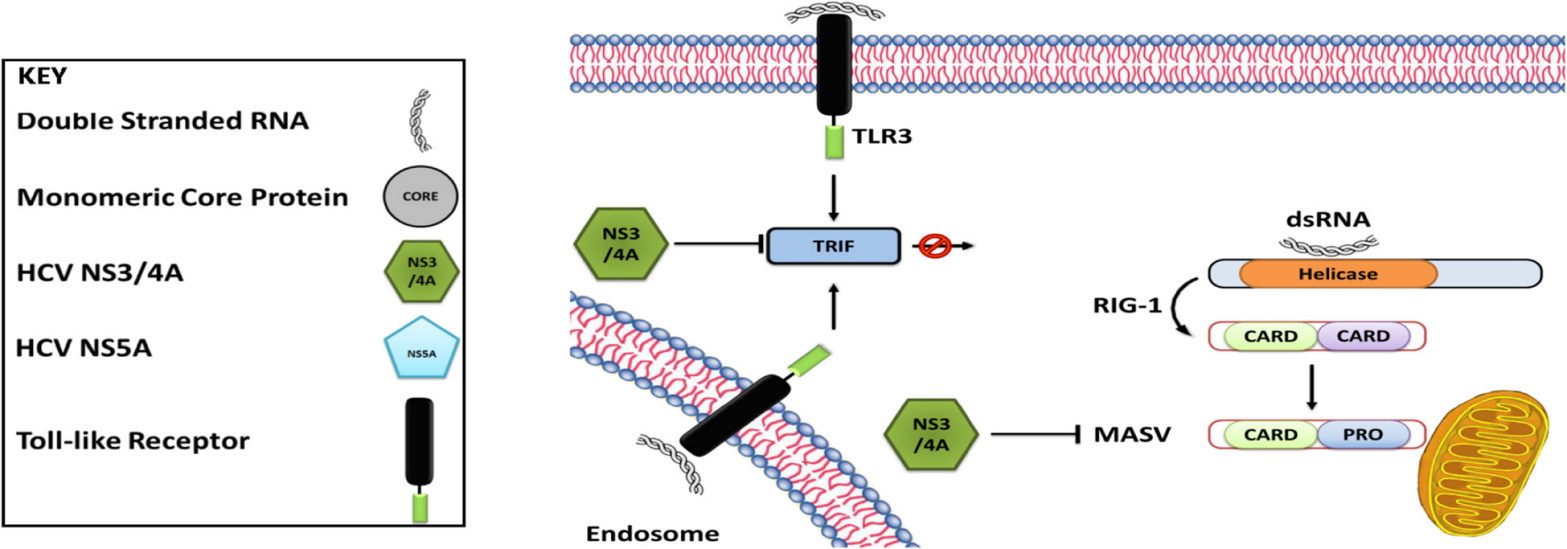
HCV NS3/4A blocks two important adapter proteins, TRIF and MAVS (Cardif) of the TLR3 signaling pathway.

TLR3 is expressed on endosomal membranes (and the plasma membranes of some cells) and senses dsRNA that is present in endosomal and/or extracellular compartments as shown in Figure 2[62]. TLR3 signaling pathway proceeds through the adaptor protein, TRIF also called TICAM-1 [58]. TRIF is the only adaptor protein that is accessible for use by TLR3. It is due to the presence of alanine in position 795 in the protruding BB loop of the TIR domain rather than the conserved proline common amongst other TLRs [58]. TRIF encompasses a TIR domain flanked by proline rich C and N terminal domains. In resting cells, TRIF does not co-localize with TLR3. It is found in diffused form throughout the cellular cytosol. dsRNA binding to TLR3 transiently recruits TRIF to localize with TLR3 at the membrane before its disassociation to form cytosolic speckle structures [63]. TRIF contributes to amino acid homology (Ser-Thr-Pro-Cys-Ser) with the HCV polyprotein NS4B/5A at the site of cleavage by NS3/4A [64,65]. Ectopically expressed TRIF is degraded in HEK293 and osteosarcoma cells expressing HCV NS3/4A. Consistent with this, polyinosinic:polycytidylic acid (poly I:C)-induced activation of IRF-3 is blocked in HeLa cells having HCV RNA replicons, and endogenous TRIF abundance is reduced in these cells [65]. Poly I:C is a synthetic dsRNA often used in studies investigating TLR3 function and signaling. However, others have not been able to demonstrate cleavage of ectopically expressed TRIF by NS3/4A [66].

Huh7 hepatoma cells, which are almost exceptional in their ability to support HCV infection in vitro, are deficient in TLR3 signaling due to a lack of TLR3 expression [67]. The absence of an HCV permissive cell line having functional TLR3/TRIF-dependent pathway has made it difficult to determine that HCV infection is sensed by TLR3. TLR3 is expressed in normal human hepatocytes in situ. Primary cultures of human hepatocytes have a robust signaling pathway, it induces the expression of interferon stimulated genes (ISGs) when stimulated by poly I:C. TLR3 is also expressed by a transduced gene in HCV permissive Huh7 cells. It senses HCV infection and establishes cellular antiviral state that restricts HCV replication. Compared to poly (I:C) stimulation, HCV infection causes a delayed TLR3-dependent interferon stimulating genes (ISG) response in hepatoma cells. This suggests that HCV may not be recognized upon viral entry. There is a requirement of HCV replication to generate an abundance of dsRNA that is sufficient to trigger TLR3 signaling. TLR3-dependent nuclear translocation of IRF-3 in HCV-infected cells suggests that dsRNA is likely to be sensed within the cell in which it is produced.

The entrance of viral RNA into the endosomal compartment and its engagement by TLR3 within infected cells is still uncertain. Conceivably, this could be accomplished through autophagy, as reported for TLR7 sensation of Vesicular stomatitis virus (VSV) infection in PDC [68]. Rab5 is an early endosome protein which colocalizes with the HCV RNA replication complex [69]. It is possible that TLR3 expression may also be localized to intracellular membranes involved in HCV RNA replication [70]. This mechanism of immune evasion blocks the expression of multiple host defense genes and contributes to persistent infections. Moreover, the ability of the protease active site to accommodate distinct substrate interactions with TRIF represents a remarkable example of RNA virus evolution. It may be the reason for the unusual hallow active site conformation that distinguishes NS3/4A from other viral proteases [60]. The cleavage of TRIF by NS3/4A characterizes a unique broad mechanism of viral immune evasion.

RIG-I senses cytosolic dsRNA. The signaling cascade involves an important adaptor protein, Cardif also called as IPS-1/MAVS/VISA [59-61,71]. This protein is another target for NS3/4A cleavage. Cardif is cleaved by NS3/4A at Cys-508 residue, 32 amino acids from the C-terminus. This results in the release of Cardif from the mitochondrial outer membrane and makes it incapable of functioning in the RIG-I signaling pathway [71,72].

#### NS3 and TLR2

TLR2 mediated innate immune signaling pathways are also stimulated by NS3 proteins. TLR2 activation involves homo or hetero dimerization with TLR1 or TLR6. NS3 stimulated TNFα and IL-10 production in human monocyte derived macrophages is impaired by TLR2, TLR1, and TLR6 knockdown. Contrary to human data; results from TLR2, TLR1, or TLR6 knockdown mice point out that the absence of TLR2 and its coreceptor TLR6, but not TLR1; prohibited NS3 protein induced peritoneal macrophage activation. In conclusion, TLR2 may use TLR1 and TLR6 coreceptors in NS3-mediated activation of macrophages and innate immunity in humans [73].

### NS3/4A and escape from the adaptive immune system

In addition to the impairment in virus-induced expression of type 1 IFNs, other cytokines induced by NS3/4A could also suppress or delay successive adaptive CD8 T cell responses. These responses are essential for HCV elimination [74,75]. Interference of virus-activated NFκB mediated responses can also promote viral persistence [76]. The proteolytic processing of components of both TLR3 and RIG-I pathways, lead to the abrogation of the cascade that activates IRF3 and NFκB. This in turn fails to induce genes that express IFN-α and -β, and additional cytokines that are crucial for stimulating other arms of the immune system.

### Studies on PBMC and cell lines for NS5A and TLR4 interactions

HCV NS5A is a hydrophilic phosphoprotein playing an important role in viral replication, modulation of cell signaling pathways and interferon response [77,78]. Its interaction with TLR4 has been suggested by various reports [79-81]. PBMCs from HCV-infected individuals show a higher expression level of TLR4 as compared to healthy individuals. HCV infection causes increased IFN-β and IL-6 secretion from B cells, particularly after LPS stimulation. The increased IFN-β and IL-6 production is attained by TLR4 induction because the introduction of small interfering RNAs against TLR4 specifcally inhibits HCV-induced cytokine production. Taking into account, all viral proteins, only NS5A causes TLR4 induction in hepatocytes and B cells. NS5A specifcally stimulates the promoter of the TLR4 gene in both hepatocytes and B cells [79].

**Figure 3:**
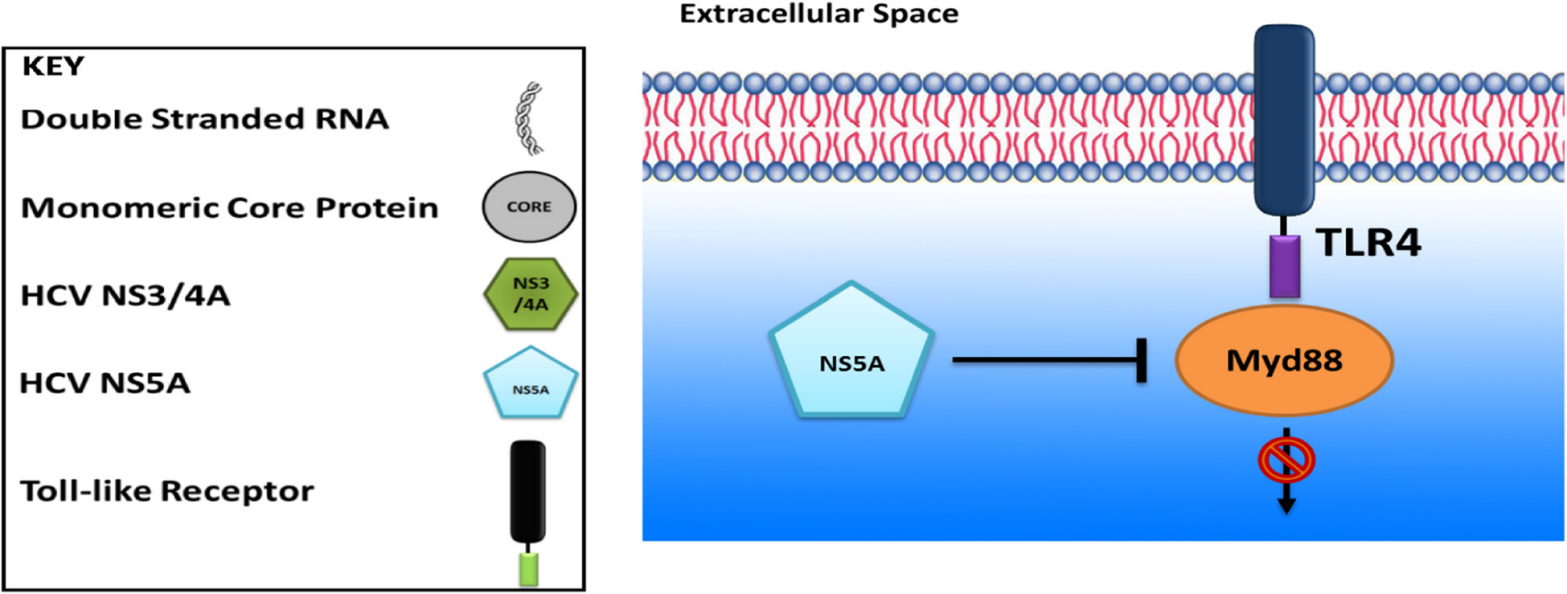
HCV NS5A blocks Myd88 of the TLR4 pathway.

On the other hand, TLR4 western blot analyses showed significant down regulation of TLR4 expression along with stable expression of HCV NS5A of genotype 1b in Huh-7 replicon cells. Huh-7 cells infected for 3 days with HCV genotype 2a (JFH1) also strongly expressed reduced levels of TLR4 in comparison with mock-infected cells. This suggests that NS5A downregulates TLR4 expression in hepatocytes. The molecular mechanisms by which NS5A decreased TLR4 expression in hepatocytes suggested that NS5A reduced TLR4 expression, at least in part, by hampering TLR4 transcription in hepatocytes. This reduction of TLR4 expression is not by its destabilization through direct interaction with NS5A. NS5A down regulates the expression of molecules involved in the formation of the TLR4 receptor complex such as MD-2, CD14. It also reduces the expression of downstream signaling molecules, MyD88, NFκB, and IRF3 [80]. Various genotypes of NS5A bind to MyD88 which is a major adaptor molecule in TLRs as shown in Figure 3. This binding hampered the recruitment of interleukin-1 receptor-associated kinase 1 to MyD88, and impaired cytokine production in response to TLR ligands. Amino acid residues 240 to 280 previously recognized as the interferon sensitivity-determining region (ISDR) in NS5A interact with the death domain of MyD88. The expression of a mutant NS5A lacking the ISDR to some extent restored cytokine production [81].

### NS5A and escape from the immune system

HCV NS5A activates TLR4 pathway [82]. NS5A potently increases the production of anti-inflammatory cytokines IL-10 and TGFβ via the TLR4 pathway. Blocking IL-10 or its receptor abrogated the NS5A-induced TGFβ secretion in a dose-dependent manner. Thus NS5A-stimulated TGFβ production is related to autocrine IL-10 release. TLR4 activation may have a role in IL-10 producing via p38 and PI3K [83]. TLR4 signaling leads to the downstream stimulation of NFkB, MAPK (p38 and JNK) and PI3K pathways [20]. PI3K is an endogenous suppressor of IL-12 production triggered by TLR4 [84]. Altogether, NS5A interacts with TLR4 on monocytes and leads to the secretion of IL-10 through p38 and PI3 kinase pathways and concurrently suppresses the production of proinflammatory IL-12. The NKG2D are activating receptors that are present on NK cells. NKG2D usually detects the presence of infectious non-self and/or stress-induced self ligands on the surface of infected cells. They are constitutively expressed on human NK and CD8 T cells [85]. Its ligands are approximately undetectable in normal tissues, but are stimulated on the cell surface by various stresses such as DNA damage, tumor transformation and intracellular infection. The importance of the NKG2D defense system is highlighted by the observation that tumors and viruses have developed several strategies for evading NKG2D-mediated recognition [86]. The overall contribution of the NKG2D pathway in the control of HCV infection is unclear [87,88]. IL-10 triggered secretion of TGFβ leads to down modulation of NKG2D expression, which in turn leads to impaired effector functions of NK cells. The functional consequences of this NKG2D reduction are associated with impaired production of IFNγ and CD107a degranulation by NK cells [82].

## Conclusion

The interaction of HCV proteins with TLRs & RIG-1 is a complex process as shown in Table 1. It stimulates various cytokines. HCV core protein interacts with TLR2 & TLR4 and stimulates various pro-inflammatory and anti-inflammatory cytokines. HCV NS3/4A protein destroys the TLR3 & RIG-1 arm of immune defense by cleaving TRIF and Cardif proteins. HCV NS5A protein enhances TLR4 transcription in PBMCs isolated from HCV patients and down regulates TLR4 transcription in cell lines. NS5A interacts with TLR4 on monocytes and induces IL10, which in turn leads to the production of TGFβ. TGFβ downmodulates NKG2D expression on NK cells and impairs NK cell function. Most of these findings were from cell lines and needs confirmation in clinical samples in order to understand the molecular mechanisms of HCV interference with the innate and adaptive immune systems.

**Table 1:** Interaction of HCV proteins with PRRs

**HCV Proteins**	**PRRs**	**Interaction of HCV proteins with PRRs**	**Escape from innate immune system**	**Escape from adaptive immune system**
**Core**	TLR2	Induction of TLR2 homotolerance	Serum derived or recombinant core does not recognize TLR2	
		Induction of TLR2 crosstolerance with TLR4	Monomeric Core recognize core	
		TLR1 or TLR6 are used as coreceptors with TLR2	Net Effect = Delayed Immune Response	
**NS3/4A**	TLR3	Block TRIF and Cadif (adapter proteins) in down-stream signaling pathways of TLR3 and RIG-1 respectively	TLR3 and RIG-1 pathways are not activated until sufficient amount of dsRNA is produced, thus delaying innate immune response	
	RIG-1			
	TLR2			
		TLR1 or TLR6 are used as coreceptors with TLR2		
**NS5A**	TLR4	Increases TLR4 expressions	Block MyD88 (adapter protein) of TLR4 signaling pathway	
			Also downregulates the expression of NFkB, MD2, CD14 and IRF3	
			Increases secretion of IL6, IFNβ, TGFβ and IL10	
			Suppresses IL12 secretion	

## Competing interests

The authors declare that they have no competing interests.

## Authors’ contributions

MI participated in data extraction, reviewing and manuscript writing; YW proof read the manuscript and helped MI in manuscript writing and publishing; MB worked on figure designing, WA worked on table, SM removed the language problems, MA gave the sketch of review article and MA provided facilitation to write the manuscript and also proofed the manuscript, SM is the PhD supervisor of MI. All authors read and approved the final manuscript.
